# A comprehensive evaluation of a novel targeted-sequencing workflow for *Mycobacterium* species identification and anti-tuberculosis drug-resistance detection

**DOI:** 10.3389/fcimb.2025.1584237

**Published:** 2025-06-09

**Authors:** Xichao Ou, Shaojun Pei, Hongru Li, Zhonghua Qin, Richard Anthony, Jichun Wang, Zexuan Song, Ruida Xing, Lixia Zhang, Chong Teng, Hui Xia, Yang Zhou, Yuanyuan Song, Yang Zheng, Shengfen Wang, Bing Zhao, Yanlin Zhao

**Affiliations:** ^1^ National Key Laboratory of Intelligent Tracking and Forecasting for Infectious Diseases, National Center for Tuberculosis Control and Prevention, Chinese Center for Disease Control and Prevention, Beijing, China; ^2^ School of Public Health, Peking University, Beijing, China; ^3^ Department of Neurology, The First Affiliated Hospital of Anhui Medical University, Hefei, China; ^4^ Center for Accurate Detection of Tuberculosis, Tianjin Haihe Hospital, Tianjin, China; ^5^ Centre for Infectious Disease Control, National Institute for Public Health and the Environment, Bilthoven, Netherlands; ^6^ Department of Science and Technology, Chinese Center for Disease Control and Prevention, Beijing, China; ^7^ Department of Tuberculosis, Beijing Dongcheng District Center for Disease Control and Prevention, Beijing, China

**Keywords:** *Mycobacterium tuberculosis*, non-tuberculous mycobacteriosis, drug resistance, multiplex PCR (polymerase chain reaction), targeted next generation sequencing (tNGS)

## Abstract

**Background:**

Although several World Health Organization–endorsed targeted next-generation sequencing (tNGS) assays exist for tuberculosis (TB) drug-resistance detection, their target selection and diagnostic accuracy vary widely. In this study, we developed a novel tNGS workflow (the TB Pro assay) and evaluated its performance in identifying *Mycobacterium* species and predicting drug resistance.

**Methods:**

The TB Pro assay was validated for identifying 10 *Mycobacterium tuberculosis* complex (MTBC) and 39 nontuberculous mycobacterial (NTM) species, as well as predicting resistance to 4 first-line and 13 second-line anti-TB drugs. The limit of detection (LOD) was determined using 11 reference strains spiked in sputum. The prediction of resistance to anti-tuberculous drugs/drug classes was compared with phenotypic drug susceptibility testing (pDST) and whole-genome sequencing (WGS) using 435 clinical isolates.

**Results:**

The assay demonstrated high sensitivity with a calculated LOD of 3.0 CFU/ml for MTB and 1.4–16.2 CFU/ml for most NTMs, except for *Mycobacterium intracellulare* with 117.9 CFU/ml. Using pDST as the reference standard, the sensitivity of the TB Pro assay for the detection of resistance ranged from 74.3% (ethambutol) to 94.4% (rifampicin), with specificity values >98% for all drugs. Compared with WGS, the sensitivity of the TB Pro assay was over 98.0% for all drugs except pyrazinamide (66.7%), and the specificity values were all nearly 100.0%. Directly on sputum, the TB Pro assay showed 100% agreement with smear- and culture-positive sputum specimens.

**Conclusions:**

The TB Pro assay represents a sensitive and specific solution for simultaneous mycobacterial identification and comprehensive drug-resistance profiling, performing robustly on both cultured isolates and direct clinical specimens.

## Introduction

In 2022, over 10 million people fell ill with tuberculosis (TB), including almost half a million with multidrug- or rifampicin-resistant TB (MDR/RR-TB) (Organization WH, 2022). Only one-third of people with MDR/RR-TB are recorded as having been diagnosed and enrolled in treatment. Drug-resistant (DR) forms of *Mycobacterium tuberculosis* complex (MTBC) have continued to increase in recent decades. Current TB treatment relies on combination drug regimens, typically guided by culture-based phenotypic drug susceptibility testing (pDST). However, pDST has critical limitations. The slow growth of MTBC delays results for weeks or months (Xu et al., 2019; Bonnet et al., 2021; Zhu et al., 2021), and the process requires stringent biosafety measures (Walzl et al., 2018). This lag often leads to ineffective treatments, resulting in lower cure rates, increased drug toxicity risks, and further selection of DR strains (Dheda et al., 2017; Pradipta et al., 2018). These challenges underscore the need for rapid, accurate diagnosis and drug-resistance detection to advance global DR-TB control (Walzl et al., 2018).

Advances in understanding DR molecular mechanisms have spurred the development of molecular diagnostics. Commercial molecular methods, such as Xpert MTB/RIF (Cepheid, Sunnyvale, CA) (Blakemore et al., 2010), can detect mutations within specific regions to predict DR, particularly for the first-line drug rifampin (Theron et al., 2014; Chakravorty et al., 2017). However, such methods miss mutations outside their limited target ranges (Akalu et al., 2022) or may misinterpret resistance-conferring variants (Fitzgibbon et al., 2021). With the development of next-generation sequencing (NGS) in recent years, sequencing-based methods can provide a comprehensive resolution of TB diagnosis, drug-resistance detection, and MTBC typing. Whole-genome sequencing (WGS) offers a sensitive method for diagnosing DR-TB (Consortium CR, the GP et al., 2018), delivering results prior to pDST (van Beek et al., 2019; Park et al., 2022). However, its high cost and requirement for specialized personnel make it impractical for low- and middle-income countries. Targeted next-generation sequencing (tNGS) technology offers a balanced solution, combining multiplex polymerase chain reaction (PCR) with NGS to simultaneously assess resistance to multiple drugs (Consortium CR, the GP et al., 2018). World Health Organization (WHO)–endorsed tNGS assays (Deeplex^@^ Myc-TB, AmPORE-TB^@^, and TBseq^@^) (Geneva: World Health Organization, 2024) demonstrate improved accuracy, though variability in target selection, PCR design, and sequencing platforms persists across platforms.

In this paper, we present the TB Pro assay (Hugobiotech Co., Ltd., Beijing, China), a novel multiplex PCR-based tNGS method. We detail its design and evaluate its performance using clinical samples and culture isolates, comparing its drug-resistance prediction sensitivity and specificity against genotypic and phenotypic DST.

## Materials and methods

### Description of TB Pro assay

#### Panel design

A more than 150-plex amplicon mix in one tube to simultaneously identify 10 MTB and 39 nontuberculous mycobacterial (NTM) strains was designed by a locally developed multiPrime with high specificity (Xia et al., 2023). According to the WHO catalogue (2022) (Walker et al., 2022), the drug-resistance gene loci were also designed in the regions targeted 20 different Mycobacterium TB genes (**Table 1**), which can detect resistance to all first-line drugs [rifampicin (RIF), isoniazid (INH), ethambutol (EMB), and pyrazinamide (PZA)], second-line drugs in group A [levofloxacin (LFX), moxifloxacin (MFX), bedaquiline (BDQ), and linezolid (LZD)], group B [clofazimine (CFZ)], and group C [amikacin (AMK), streptomycin (STM), ethionamide (ETH), and prothionamide (PTO)], cycloserine (CYC), kanamycin (KAN), and capreomycin (CAP) (Geneva: World Health Organization, 2021). The specific mixtures of target primers were provided in the Tuberculosis and Nontuberculous Mycobacteria Identification and Drug Resistance Genes Detection Reagent Kit (YG-026-48; Hugobiotech Co., Ltd., Beijing, China).

**Table 1:** Drug resistance-associated targets designed by TB Pro assay.

Genomic targets	Drugs	Genomic targets	Drugs
rpoB*	rifampicin	*gid*	streptomycin
inhA*	isoniazid, ethionamide, Prothionamide	rpsL*	streptomycin
*KatG*	isoniazid	*rrs*	streptomycin
*pncA*	pyrazinamide	*ethA*	ethionamide, Prothionamide
*embA*	ethambutol	eis*	kanamycin, amikacin
embB*	ethambutol	*tlyA*	capreomycin
gyrA*	levofloxacin, moxifloxacin	*alr*	Cycloserine
*gyrB*	levofloxacin, moxifloxacin	*folC*	para-Aminosalicylic Acid
Rv0678*	bedaquiline, clofazimine	*ribD*	para-Aminosalicylic Acid
rplC*	linezolid	*thyA*	para-Aminosalicylic Acid

*These genes include all the Groups 1 and 2 mutations in the WHO catalogue (2023).

#### Multiplex PCR and library construction

**Figure 1:**
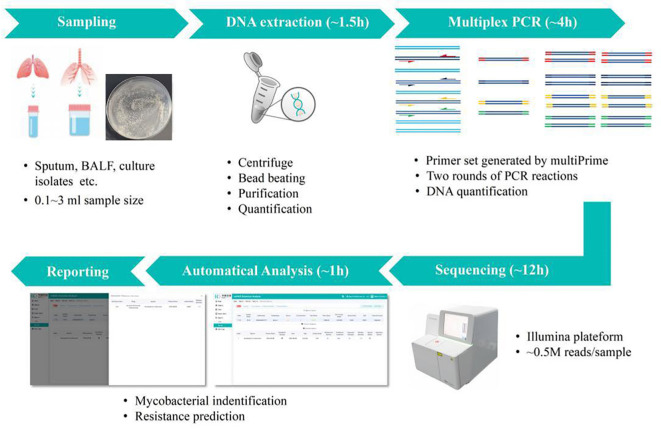
Overview of TB Pro assay workflow. The assay relies on deep sequencing of more than 150-plex amplicon mix in one tube, for simultaneous 10 *Mycobacterium tuberculosis* (MTB) strains and 39 nontuberculous mycobacterial (NTM) species identification, and prediction of 20 drug resistance-associated genes of MTBC strains. Samples can be pooled and analyzed in a single bench on different Illumina^®^ platforms, with a maximum 24-h turnaround time to generate final report.

The DNA of the samples was extracted and purified using the Nucleic Acid Extraction Reagent kit (YG-023-48; Hugobiotech Co., Ltd., Beijing, China) and Nucleic Acid Purification Reagent kit (YG-024-48; Hugobiotech Co., Ltd., Beijing, China). Subsequently, all multiplex amplifications were performed in one tube, and detection of the targeted sequences was done on a TGreat Expert gradient thermal cycler (TIANGEN, Beijing, China). The protocol of PCR and library construction was followed by the sequencing reaction universal reagent kit protocol (YG-025-48; Hugobiotech Co., Ltd., Beijing, China). The quantified PCR library was sequenced on the Nextseq 550 platform (Illumina, San Diego, USA) in this study. In parallel with the clinical samples, positive control and negative control (non-template control, NTC) were also included for each batch of NGS run with the same procedure and bioinformatics analysis. The workflow is shown in **Figure 1**.

#### Bioinformatic analysis

After removing the adapter, low-quality, low-complexity, and shorter reads of <35 bp (Chen et al., 2021) of high-quality sequencing data were retained. Next, human reads were filtered out by mapping reads to the human reference genome (GRCh38) using Bowtie 2 (Langmead and Salzberg, 2012). The remaining cleaned data was aligned to the multi-FASTA reference library and antibiotic resistance database using Burrow-Wheeler Aligner software (Li and Durbin, 2009). The sequencing data was automatically analyzed on the TB Pro Web Application, which generated the final report in one hour.

### Dilutions and spiking in sputum for determining the limit of detection

Eleven reference strains, *Mycobacterium tuberculosis* H37Rv (ATCC 27294), *Mycobacterium tuberculosis* variant bovis (ATCC 19210), *Mycobacterium tuberculosis* variant BCG (ATCC 27289), *Mycobacterium avium* (ATCC 25291), *Mycobacterium abscessus* (ATCC 19977), *Mycobacterium intracellulare* (ATCC 13950), *Mycobacterium kansasii* (ATCC 12478), *Mycobacterium malmoense* (ATCC 29571), *Mycobacterium xenopi* (ATCC 19250), *Mycobacterium marinum* (ATCC 927), and *Mycobacterium fortuitum* (ATCC 6841) were preserved in the National Tuberculosis Reference Laboratory (NTRL) (Xu et al., 2024). The limit of detection (LOD) of the TB Pro assay was determined by spiking the above serial dilutions of strains of quantified colony-forming units (CFU) into *Mycobacterium-*negative sputum samples and testing each sample according to a standard TB Pro process. Analytical sensitivity tests were performed by spiking known numbers of CFU in sputum in a dilution series from 10^4^ CFU/ml to 0 CFU/ml. The assay LOD was defined as the lowest number of CFU that, when spiked into 1 ml of sputum, would result in the detection of *Mycobacterium* species 95% of the time that a test was performed. Probit analysis was performed to calculate the LOD using SPSS software.

### Clinical isolates collection and phenotypic DST

The clinical MTB strains were obtained from the NTRL in China. All strains were subcultured on Löwenstein-Jensen medium and incubated at 37°C for 3 weeks. Several colonies were selected, and phenotypic DST [minimal inhibitory concentration (MIC)] was performed using the broth microdilution method. The colonies were inoculated into test tubes containing saline-Tween and glass beads using a sterile loop (Consortium CR, the GP et al., 2018). After vortexing and settling the inoculum, a 0.5 McFarland standard equivalent was prepared using an ultrasonic milling instrument. Suspensions were diluted 100-fold by adding 100 μl of the 0.5 Mc suspension to 10 ml of Mueller–Hinton broth with/without OADC. Aliquots of 100 μl of the standard 1.5 × 10^5^ CFU/ml inoculum were distributed to each well using a semi-automated Sensititre™ Auto-inoculator (Thermo Fisher Scientific Inc., Waltham, MA, USA). The Sensititre^®^ UKMYC6 plate, with 12 anti-TB drugs, including RIF, INH, EMB, LFX, MFX, BDQ, LZD, CFZ, AMK, ETH, KAN, and delamanid (DLM), was sealed and incubated at 37°C. The MIC was defined as the lowest concentration without considerable visible bacterial growth compared with the positive controls, as determined using the VizionTM Digital viewing system. MTB H37Rv (ATCC 27294) was used for quality control for each batch. Based on the critical concentration recommended by the WHO (Walker et al., 2022), the breakpoints for INH, RIF, EMB, and MFX were 0.1, 0.5, 4, and 1 μg/ml, respectively (**Supplementary Table S1**). Inconclusive results for EMB were defined as an MIC of 4 μg/ml that does not correlate with either a susceptible or resistant result.

### Whole-genome sequencing analysis of clinical isolates

Genomic DNA of the 435 clinical MTB strains was extracted from the colonies, which were incubated on solid media for pDST as mentioned above, and was subjected to WGS using the Illumina HiSeq 2000 platform with a paired-end strategy, as described previously (He et al., 2022). The remaining 435 DNA templates were sequenced by TB Pro assay following the standard TB Pro workflow. The WGS analysis was performed using the Clockwork pipeline with default parameters originally developed for the CRyPTIC Consortium by a research team at the European Bioinformatics Institute (Walker et al., 2022). Mutations in genes in the proline–glutamic acid/proline–proline–glutamic acid family and in regions with repetitive sequences were excluded from the phylogenetic analysis. A maximum likelihood phylogenetic tree was constructed using IQTREE with 1000 bootstrap replicates and a general time-reversible (GTR+G) model of nucleotide substitution. The phylogenetic tree was visualized and modified using iTOL (v6.4.3). Lineage and sub-lineage strains were identified using a fast-lineage caller (v1.0). WGS predictions of phenotypic susceptibility were based on the final confidence grading of mutations within or upstream of drug resistance-related genes, isolates with resistance-conferring mutations assigned to Groups 1 (associated with resistance) and 2 (associated with resistance-interim) were considered genetically drug resistant (Walker et al., 2022).

### Direct TB Pro performance on sputum samples

Totally, 67 clinical sputum samples were provided by NTRL. Briefly, processed primary sputa were stained using Auramine O and examined under standard smear LED fluorescent microscopy grading of acid-fast bacilli (AFB). Another sputum from the same patient, which was simultaneously collected, was used to perform the Xpert MTB/RIF test and liquid culture (MGIT 960). The remaining sputa were incubated for 10 min at 95°C, and then DNA was extracted and purified from the remnant according to the instruction of the Nucleic Acid Extraction Reagent kit (YG-023-48; Hugobiotech Co., Ltd., Beijing, China). The quality and concentration of DNA samples were monitored by Qubit Fluorometer 4.0 (Thermo Fisher Scientific, MA, USA) and analyzed with the TB Pro assay using the NextSeq platform.

### Statistical analysis

The sensitivity and specificity of the TB Pro assay for RIF, INH, EMB, LFX, and MFX resistance detection were calculated using the exact Clopper-Pearson confidence interval with phenotypic DST and WGS as the gold standards. The diagnostic performance for STM, ETH, and PZA resistance detection was calculated using the exact Clopper-Pearson confidence interval, with WGS as the gold standard. SPSS (IBM Inc. 2019, Armonk, NY, USA) was used to calculate kappa statistics. The consistency of the test results obtained using the two methods was interpreted by the κ value as follows: 0.81–1 indicated nearly perfect, 0.61–0.80 indicated substantial consistency, 0.41–0.60 indicated moderate consistency, 0.21–0.40 indicated fair consistency, and 0.0–0.20 indicated slight consistency (Tang et al., 2015).

## Results

### Laboratory validation of the TB Pro assay

#### Limits of detection

**Figure 2:**
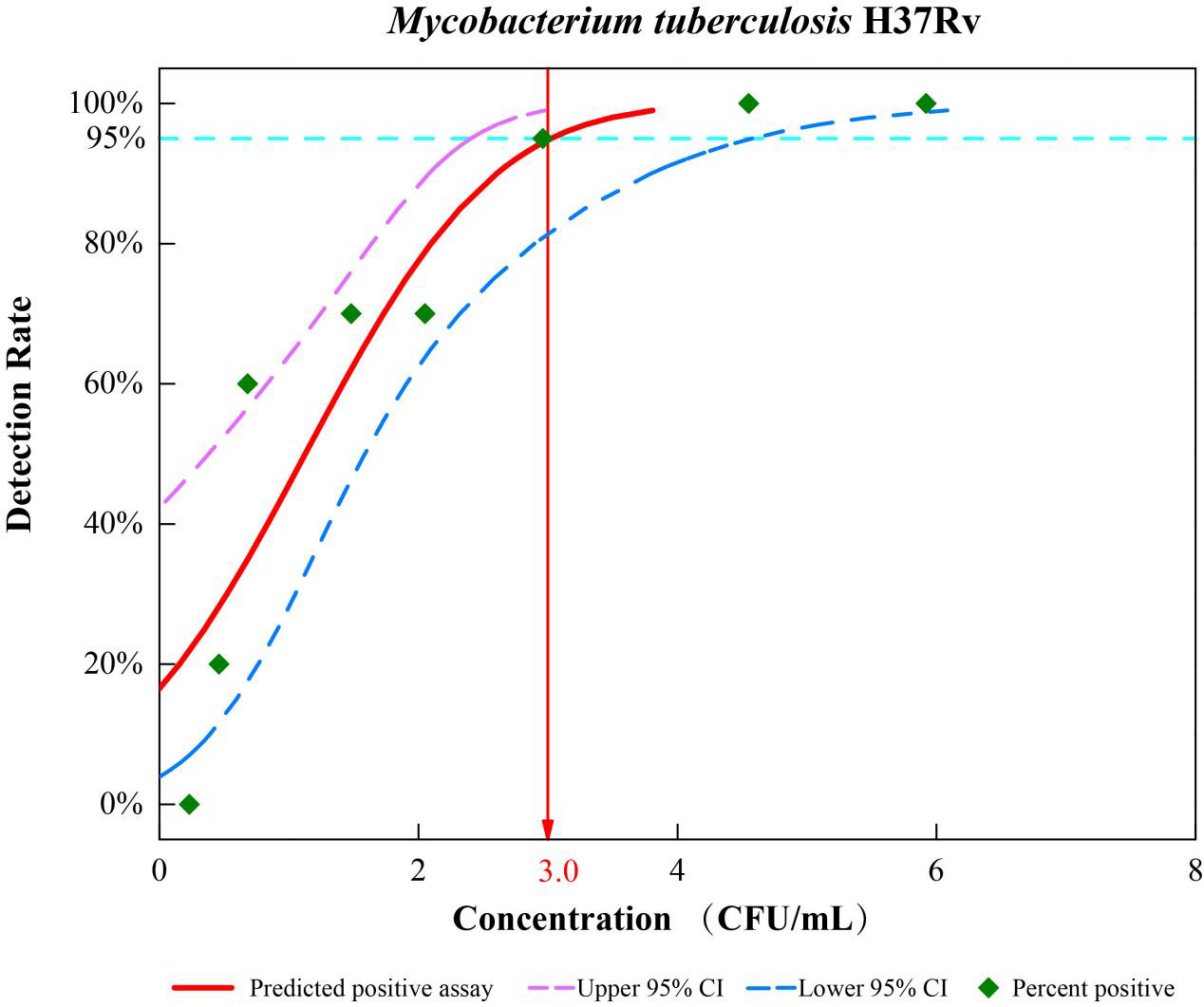
Limits of detection (LOD) of the TB Pro assay. Analytical sensitivity tests were performed by spiking known numbers of H37Rv in sputum in a dilution series from 10^4^ CFU/ml to 0 CFU/ml, with 20 replicates for each cell concentration, and probit analysis was performed to calculate the LOD using SPSS software.

The comparative analytical LODs of TB Pro were tested by spiking serial dilutions of quantified CFU into sputum samples. We found that the TB Pro assay has a calculated LOD of 3.0 CFU/ml of sputum (95% CI, 2.4–4.6 CFU/ml) (**Figure 2**). The high sensitivity of TB Pro was also evident at lower LOD concentrations, with 70% of the samples tested at 1.5 CFU/ml still positive by TB Pro. Known number of *M. bovis* and *M. bovis* BCG CFU were spiked into sputum samples, resulting in a nearly identical LOD estimate. LOD for NTM identification showed the analytical sensitivity from 1.4 to 16.2 CFU/ml, except for *M. intracellulare* with 117.9 CFU/ml (**Supplementary Figures S1, S2**).

#### Precision and interference

Specimens were spiked with different *Mycobacterium* species mixtures and performed by different people each day to ensure the precision estimates included all possible sources of variation. Precision duplicate measurements of individual microorganisms showed no correlation to either personnel or reagent batches (**Supplementary Table S2**). The addition of exogenous DNA at high, medium, and low levels did not impact qualitative detection. Additionally, neither albumin, glucose, nor drugs (rifampicin and clarithromycin) affected the TB Pro detection results.

### Diagnostic efficacy of the TB Pro assay compared with phenotypic DST

According to the phenotypic DST results, 24.6% (107 of 435), 52.2% (227 of 435), 19.3% (84 of 435), 20.9% (91 of 435), and 8.0% (35 of 435) of the strains were resistant to RIF, INH, MXF, LXF, and EMB, respectively (**Supplementary Table S3**). In addition, inconclusive results were obtained for 35 strains for EMB resistance detection because the MIC was 4 μg/ml. The overall performance of TB Pro, including the sensitivity, specificity, accuracy, and κ value for each drug, is shown in **Table 2**. Our results show that TB Pro can predict MTB drug resistance with high sensitivity and specificity. The sensitivity ranged from 74.3% (EMB) to 94.4% (RIF); all specificity values were >98%. All the drugs had κ values >0.8.

**Table 2:** Diagnostic efficiency of the TB Pro assay compared with that of phenotypic drug susceptibility testing.

Drugs	pDST resistant			pDST susceptible			Sensitivity % [95% CI]	Specificity % [95% CI]	κ value
	TB Pro (*n*)		Total (*n*)	TB Pro (*n*)		Total (*n*)			
	R	S		R	S				
RIF	102	5	107	3	325	328	95.3 [88.9–98.3]	99.1 [97.1–99.8]	0.95
INH	194	33	227	4	204	208	85.5 [80.0–90.0]	98.1 [94.8–99.4]	0.83
EMB	26	9	35	0	365	365	74.3 [56.4–86.9]	100.0 [98.7–100.0]	0.84
MFX	78	6	84	5	346	351	92.8 [84.5–97.0]	98.6 [96.5–99.5]	0.92
LFX	80	11	91	3	341	344	87.9 [79.0–93.5]	99.1 [97.2–99.8]	0.89
ETH	32	5	37	23	375	398	86.5 [70.4–94.9]	94.2 [91.3–96.2]	0.66

pDST, phenotypic drug susceptibility testing; RIF, rifampin; INH, isoniazid; EMB, ethambutol; MXF, moxifloxacin; LFX, levofloxacin; ETH, ethionamide.

Inconsistencies were observed between pDST and TB Pro assay results for certain strains (**Supplementary Table S4**). Of the 107 phenotypic RIF-resistant strains, 5 were RIF-sensitive using the TB Pro assay. The TB Pro assay missed one *rpoB*_p.Lys446Thr, which was correctly called by WGS. No mutations were detected by WGS in the other four phenotypic RIF-resistant strains. Three phenotypic RIF-susceptible strains were identified as RIF-resistant using TB Pro, and all three strains carried borderline *rpoB* mutations (*rpoB*_p.His445Asn and *rpoB*_p.Leu430Pro) (Xia et al., 2022). Discrepancies in 33 phenotypic INH-resistant strains were also observed, with WGS identifying two mutations (*katG*_p.Trp149* and *katG*_p.Trp204*) not covered in the TB Pro assay. Fourteen INH-discordant strains had no known drug-resistance mutations, and the remaining 16 strains carried rare *katG* mutations or mutations upstream of *ahpC*, listed as Group 3 (uncertain significance) in the 2021 and 2023 WHO catalogue. Further, four phenotypic INH-susceptible strains were identified as INH-resistant using the TB Pro assay, and mutations were all consistent with WGS analysis. Additionally, LXF and MXF resistance predictions by TB Pro did not always align with WGS findings, and some strains with *gyrA* mutations showed phenotypical susceptibility despite genotypic resistance indicated by TB Pro and WGS. Some strains showed phenotypic LXF/MXF resistance but without corresponding DR mutations.

### Comparison of the diagnostic efficacy of the TB Pro assay and WGS

**Figure 3:**
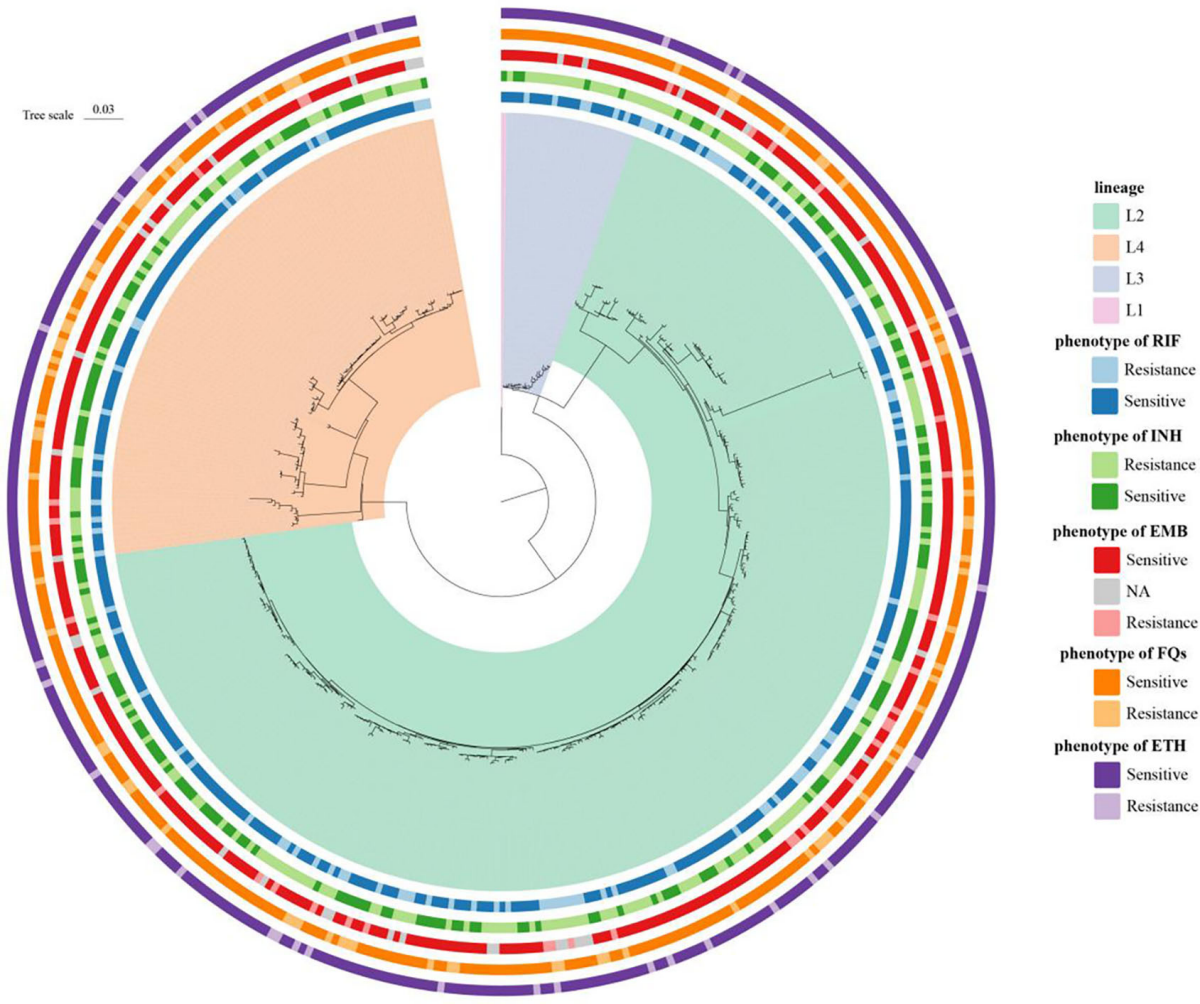
Phylogenetic construction of *Mycobacterium tuberculosis* strains (*n* = 435) in China. Maximum-likelihood phylogenetic tree showed four lineages, including the most prevalent MTB lineages worldwide. The circles show the drug sensitivity profiles, with lighter colors representing isolates being resistant to tested drugs.

Among 435 MTB isolates, 69.2% (301 of 435), 5.5% (24 of 435), and 25.1% (109 of 435) were assigned to lineages 2 (East Asian genotype), 3 (Indian and East African genotypes), and 4 (Euro-American genotype) (**Figure 3**), respectively, which indicated high-genetic diversity, including the most prevalent MTB lineages worldwide (Coscolla and Gagneux, 2014).

Subsequently, we analyzed genotypic resistance for the anti-TB drugs. The total resistance rates were 24.6% (107 of 435), 46.2% (201 of 435), 10.6% (46 of 435), 4.8% (21 of 435), 18.8% (82 of 435), 36.8% (160 of 435), and 12.9% (56 of 435) for RIF, INH, EMB, PZA, FQs, STM, and ETH, respectively. Six genotypically resistant strains for KAN & AMK were detected by WGS and TB Pro, while CFZ, BDQ, and LZD mutations were not detected neither by WGS or TB Pro. Compared with WGS results, the sensitivity, specificity, and κ values of the TB Pro assay for detecting resistance were 98.1%, 100.0%, and 0.99 for RIF; 98.5%, 100.0%, and 0.99 for INH; 100.0%, 100.0%, and 1.00 for EMB; 66.7%, 100.0%, and 0.79 for PZA; 100.0%, 99.7%, and 0.99 for FQs; 98.1%, 100.0%, and 0.98 for STM; and 98.2%, 100.0%, and 0.99 for ETH, respectively (**Table 3**). Inconsistencies were observed between WGS and the TB Pro assay in 17 strains (**Supplementary Table S5**). Most miss-detected mutations are not targeted by the TB Pro assay, seven of which are related to PZA resistance. There were also individual mutations that were undetected because the mutation rate was below the threshold.

**Table 3:** Phenotype predictions by TB Pro assay of DNA from 435 *versus* phenotype predictions by WGS.

Drugs	Genotypically resistant			Genotypically susceptible			Sensitivity% [95% CI]	Specificity% [95% CI]	κ value
	TB Pro (*n*)		Total (*n*)	TB Pro (*n*)		Total (*n*)			
	R	S		R	S				
RIF	105	2	107	0	328	328	98.1 [92.7–99.7]	100.0 [98.6–100.0]	0.99
INH	198	3	201	0	234	234	98.5 [95.3–99.6]	100.0 [98.0–100.0]	0.99
EMB	46	0	46	0	389	389	100.0 [90.4–100.0]	100.0 [98.8–100.0]	1
PZA	14	7	21	0	414	414	66.7 [43.1–84.5]	100.0 [98.8–100.0]	0.79
FQs	82	0	82	1	352	353	100.0 [94.4–100.0]	99.7 [98.2–100.0]	0.99
STM	157	3	160	0	275	275	98.1 [94.2–99.5]	100.0 [98.3–100.0]	0.98
ETH	55	1	56	0	379	379	98.2 [89.2–99.9]	100.0 [98.7–100.0]	0.99

WGS, whole genome sequencing; RIF, rifampin; INH, isoniazid; EMB, ethambutol; FQs, fluoroquinolones; STM, streptomycin; PZA, pyrazinamide; ETH, ethionamide.

### Heteroresistance detection ability of the TB Pro assay

Notably, TB Pro is able to identify heteroresistance, which refers to the coexistence of both the wild-type and mutant alleles for the same codon. In this study, 54 of 435 isolates presented heteroresistance with the mutant allele frequency <90%, involving in the *katG*, *inhA*, *embB*, *pncA*, *gyrA*, *gyrB*, *rpsL*, and *rrs* genes (**Supplementary Table S6**). There was complete concordance between TB Pro’s predictions of susceptible MFX and INH and those (alleles at a frequency exceeding 90.0%) determined by WGS analysis, with the exception of EMB. Heteroresistance was mainly observed in the *gyrA* gene, with variant frequencies varying between 10% and 88%. Notably, 90% (18 of 20) of minority resistance variants (with frequencies <25%) accurately predicted phenotypic resistance to fluoroquinolones.

### Accuracy of TB Pro assay on clinical sputum

**Figure 4:**
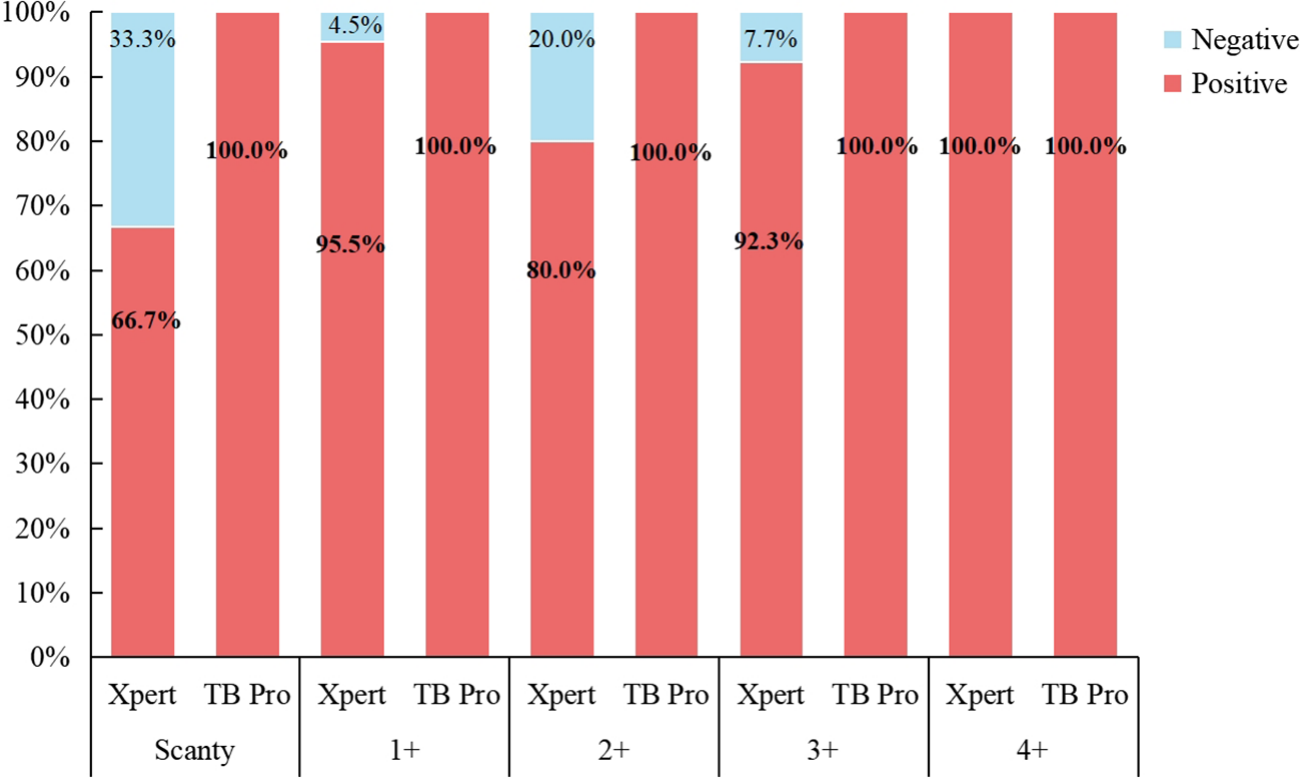
The consistency between Xpert and TB Pro assay in species identification compared against smear microscopy results.

Among the 67 smear-positive clinical samples, 63 (94.0%) were genotyped as MTB DNA by TB Pro assay, one was genotyped as *M. bovis*, and the other five were NTM species. Fifty-six of these smear-positive samples grew MTBC, while five grew *M. avium*, *M.abscessus*, *M. intracellulare*, and *M. kansasii*, respectively (**Supplementary Table S7**). Compared with 61 culture results, TB Pro showed 100% concordance. One sample was cultured for MTBC but was genotyped as *M. bovis* by TB Pro. These six culture-negative samples were all genotyped by TB Pro. In addition, all of the 67 samples were performed GeneXpert MTB/RIF assay and 59 (88.0%) were detected as containing MTBC DNA. One of the Xpert-positive results was genotyped as *M. bovis* by TB Pro. Four of eight Xpert-negative samples were detected as MTB by TB Pro assay, which were all verified by culture-positive results. Interestingly, one Xpert-positive sample with semi-quantitative “very low” was cultured with a discrepant result as *M.abscessus*, while the sample was identified as a mix of MTB and *M.abscessus* by TB Pro. Summarizing the above results, accuracy of species typing for TB pro was 100% (**Figure 4**).

Among the 61 MTBC culture-positive specimens, 55 generated successful pDST results, including 40 strains with pan-susceptible phenotype (**Supplementary Table S7**). Agreement on resistance prediction of the TB Pro assay was over 90.0% for all applicable drugs, except for INH (85.7%). RIF, INH, PZA, FQs, ETH, and PTO resistance were additionally predicted by TB Pro in eight susceptible strains, which presents the potential value for tNGS to make up for the limited availability of phenotypic DST testing of primary clinical specimens. For samples with discrepancies between pDST and TB Pro, we performed WGS on the cultures. The results indicated that, aside from the missed detection of mutations in *pncA*, the identification of other drug resistance genes at the Groups 1 and 2 loci was consistent between WGS and TB Pro (**Supplementary Table S8**). Overall, these results demonstrated that TB Pro can accurately identify susceptible and resistant forms of MTBC directly from clinical specimens.

## Discussion

MTB infection is recognized among infectious diseases as the leading cause of death. Additionally, the incidence and prevalence rate of NTM disease are increasing rapidly (Johansen et al., 2020). MTB and some NTM infections are clinically indistinguishable, but they differ significantly in terms of treatment outcomes and antibiotic susceptibilities (Griffith et al., 2007; Haworth et al., 2017). Therefore, optimal treatment strategies rely on early species identification, making it clinically relevant to develop a method for rapidly and accurately detecting major *Mycobacterium* species. The current study describes the development of a new platform (TB Pro assay) that provides extreme sensitivity to identify species with the minimum bacterial loads from 1.4 to 16.2 CFU/ml, which is significantly lower than any previous rapid diagnostic tests (Jouet et al., 2021; Murphy et al., 2023).

Importantly, with the increasing number of DR-TB worldwide, early diagnosis and detection of drug sensitivity are essential for TB control (Walzl et al., 2018). tNGS represents a sensitive, reliable, and accurate method for detecting DR-TB and offers great potential to provide comprehensive resistance detection matched to modern treatment regimens. In the study, multiple PCR primers of the TB Pro assay were designed based on the multiPrime platform (Xia et al., 2023), aiming to balance mismatch tolerance and maximize compatibility and specificity. The high-throughput sequencing downstream further evaluates both the counts and the sequence information of the amplicons. Due to the absence of human or other microbial nucleic acid in the amplified products, a single sample library only requires approximately 0.5M reads (in contrast to the typical requirement of at least 20M reads for metagenomic NGS), thereby greatly improving detection throughput and reducing sequencing costs. Additionally, the multiplex PCR technique enables the incorporation of over 1,000 primers within a single reaction tube (Chae et al., 2017; Kim et al., 2018), allowing for the flexible inclusion of new targets as required in clinical settings by introducing novel primers, all while maintaining nearly unchanged costs. Compared with the currently WHO-endorsed assays, for example, the Deeplex assay, TB Pro has a lower LoD of MTB identification (3.0 CFU/ml vs. 100 genomes/ml) (Jouet et al., 2021) and a shorter turnaround time (24h vs. 40h), which can meet the clinical requirement of rapid diagnosis and targeted treatment. However, due to differences in design rules of mutation sites and library construction methods, TB Pro mainly focuses on identifying clear/known DR mutation loci, while Deeplex can discover some new mutations in DR genes, making the latter more scientifically valuable for research. In summary, we believe that the TB Pro assay could be a good sensitivity, scalability, and cost-effectiveness tool for early detection of TB and should be considered for implementation in public health and clinical laboratories.

The global incidence of NTM infections has been steadily increasing across most regions (Prevots et al., 2023). Unlike TB, NTM infections are not notifiable diseases in most countries. Due to the high similarity of clinical symptoms between NTM and TB, these patients are frequently misdiagnosed with MTBC infection. However, NTM infections require specific drug treatment regimens, and such misdiagnoses often lead to treatment delays that adversely affect patient outcomes. Emerging epidemiological data reveal distinct species-specific trends, underscoring the critical need for precise identification in NTM surveillance and clinical management. Current NTM diagnosis primarily relies on culture and species-level biochemical identification but faces significant limitations, including restricted coverage of conventional PCR assays, prolonged turnaround times for 16S rRNA sequencing, and the cost-prohibitive nature of mNGS. In this context, TB Pro offers valuable preliminary NTM identification to support clinical decision-making, though with important caveats. The assay demonstrates variable sensitivity across NTM species, with *M. intracellulare* showing a notably higher LOD (117.9 CFU/ml) compared to other NTM species. These performance characteristics emphasize that while TB Pro results can provide early diagnostic guidance, they should be interpreted as presumptive findings requiring confirmation through standard culture methods. This two-tiered approach balances the need for rapid clinical information with the rigorous requirements for definitive NTM diagnosis and epidemiological monitoring.

Phenotypic DST remains the reference standard for detecting resistance to most anti-TB medicines (MacLean et al., 2023). In the current study, most cultured isolates yielded concordant susceptibility profiles between pDST and the TB Pro assay. For rifampicin, three discordant strains carried borderline *rpoB* mutations (Xia et al., 2022) that confer intermediate resistance through subtle structural changes in RNA polymerase, producing MIC values near the critical concentration that conventional DST may miss. These mutations exhibit partial resistance with variable detection across testing conditions, unlike definitive resistance mutations. The absence of canonical resistance mutations in other discrepant cases suggests the genotype-phenotype relationship for rifampicin resistance determining region (RRDR)–adjacent rpoB mutations requires further elucidation. For isoniazid, phenotype-genotype agreement did not perform well. The mutations of the *ahpC* gene were not included in our targets because more than 200 mutations are endorsed in the WHO catalogue (Group 3) and are hard to balance the compatibility and specificity with other primers. Previous studies demonstrated mutations in *ahpC* and the *ahpC*-*oxyR* intergenic regulatory region have been identified in INH-resistant isolates, and these *ahpC* mutations could produce a compensatory effect with non-315 amino acid mutations conferring high-level resistance (Sreevatsan et al., 1997; Liu et al., 2018). This represents a key limitation of our assay, necessitating supplemental testing for ahpC variants in TB Pro-reported INH-susceptible cases, with plans to address this gap through WHO-guided updates. Discordant results for ethambutol, moxifloxacin, and ethionamide likely reflect inherent limitations of pDST as either an imperfect reference method or one validated exclusively for MGIT medium (Walker et al., 2015; Geneva: World Health Organization, 2018). Regarding pyrazinamide, while molecular methods are preferred given pDST’s unreliability, TB Pro cannot currently serve as a frontline PZA resistance assay due to technical constraints involving the pncA gene’s excessive length and its incompatibility with multiplex PCR amplification requirements.

Crucially in this respect, the TB Pro assay presented a high degree of accuracy for prediction of drug resistance with an efficiency close to WGS. The main reason for inconsistency with WGS results is that TB Pro did not design targets around these mutations. As per the latest recommendations from WHO (Geneva: World Health Organization, 2023), we reduced the threshold of all variants to estimate the impact of minority variants. As expected, lowering the cutoff for calling Groups 1 and 2 mutations from 75% to 25% increased the combined sensitivity of these mutations for predicting MFX-resistant phenotypes by approximately 37.0%. In addition, when we further lower the cutoff for calling variants to an allele frequency of 10%, 90.0% of resistance predictions were confirmed as true results when validated against both WGS and pDST reference standards. These results in the study confirmed published evidence that heteroresistance plays an important role in FQ resistance (Lakshmi et al., 2015; Brankin and Fowler, 2023).

For clinical implementation, several factors require consideration. While the 24h workflow is faster than culture-based DST, the TB Pro assay demands NGS infrastructure and trained personnel, potentially limiting use in resource-limited settings. While reagent costs are lower than WGS, the per-test price remains higher than LPAs, though this is partially offset by eliminating cascade testing. Performance wise, the assay shows 94% concordance with culture for smear-positive samples, though sensitivity may decline with paucibacillary specimens. Strategies such as hub-and-spoke testing models and automated reporting could improve feasibility. These practical considerations, combined with the assay’s technical advantages in sensitivity (3.0 CFU/ml LOD) and comprehensive resistance profiling, position TB Pro as a promising tool for public health laboratories with adequate infrastructure, particularly for programmatic DR-TB surveillance and complex diagnostic cases.

The present study has several limitations. First, the number of tested smear-positive samples was limited. Although TB Pro demonstrated a significantly higher detection rate than AFB smear and culture in a recent study using BALF samples (Wu et al., 2023), further validation of its analytical sensitivity and specificity in larger, multicenter cohorts representing diverse geographic regions is still needed. Second, pDST data of PZA, STM, and ETH were hard to perform or unreliable in solid media (Zhang et al., 2002; Chedore et al., 2010; Lakshmi et al., 2015), so we did not compare the accuracy between the TB Pro assay and pDST. However, our results showed a close match in susceptibility predictions between TB Pro and WGS analysis. Third, among the 435 strains analyzed, resistant phenotypes for second-line drugs, including new drugs (bedaquiline and delamanid) and repurposed drugs (linezolid and clofazimine), were underrepresented. Future studies will prioritize the collection of such resistant strains (e.g., through international collaborations) to validate resistance predictions globally. Finally, we found that multiple mutations seemed to generate high-level resistance, but due to the limited data, we did not categorize and analyze them.

In conclusion, the results of the system evaluation demonstrated the potential of the TB Pro assay to reliably identify *Mycobacterium* species and detect anti-tuberculous drug resistance from culture or directly from clinical specimens. This assay can be performed as a fast molecular method to guide personalized TB treatment and covers the needs of many clinical laboratories at local/regional or nationally centralized levels with a turnaround time of <24h, significantly reducing the time for generating extended DST reports. While the capacity of the TB Pro assay to detect the new/repurposed drugs, including bedaquiline, clofazimine, and delamanid/pretomanid, needs to be further evaluated.

## Data Availability

The datasets presented in this study can be found in online repositories. The names of the repository/repositories and accession number(s) can be found in the article/**Supplementary Material**.
