# Supplementary material for: A comprehensive evaluation of a novel targeted-sequencing workflow for Mycobacterium species identification and anti-tuberculosis drug-resistance detection
Source: Front Cell Infect Microbiol. 2025 Jun 9;15:1584237. doi: 10.3389/fcimb.2025.1584237 (PMC12183266; doi:10.3389/fcimb.2025.1584237)
Supplement: Supplementary file 1 [file Table1.docx]

***Supplementary Material***

**A comprehensive evaluation of a novel targeted-sequencing workflow for *Mycobacterium* species identification and anti-tuberculosis drug resistance detection**

Xichao Ou ^a#^, Shaojun Pei ^a, b#^, Hongru Li ^c#^, Bing Zhao ^a^, Jichun Wang ^d^, Zexuan Song ^a^, Ruida Xing ^a^, Lixia Zhang ^e^, Chong Teng ^f^, Hui Xia ^a^, Yang Zhou ^a^, Yuanyuan Song ^a^, Yang Zheng ^a^, Shengfen Wang ^a^, Zhonghua Qin ^e*^, Richard Anthony ^g*^, Yanlin Zhao ^a*^

^a^ National Key Laboratory of Intelligent Tracking and Forecasting for Infectious Diseases, National Center for Tuberculosis Control and Prevention, Chinese Center for Disease Control and Prevention, Beijing, 102206, China

^b^ School of Public Health, Peking University, Beijing, 100191, China

^c^ Department of Neurology, the First Affiliated Hospital of Anhui Medical University, Hefei, 230022, China

^d^ Department of Science and Technology, Chinese Center for Disease Control and Prevention, Beijing 102206, China

^e^ Center for Accurate Detection of Tuberculosis, Tianjin Haihe Hospital, Tianjin, 300350, China

^f^ Department of Tuberculosis, Beijing Dongcheng District Center for Disease Control and Prevention, Beijing 100050, China

^g^ National Institute for Public Health and the Environment, BA Bilthoven, 3721BA, Netherlands

**#The authors contributed to this work equally**

***Authors for correspondence:** Yanlin Zhao, National Key Laboratory of Intelligent Tracking and Forecasting for Infectious Diseases, National Center for Tuberculosis Control and Prevention, Chinese Center for Disease Control and Prevention, Beijing, 102206, China. E-mail: zhaoyl@chinacdc.cn. phone numbers: +861058900517; Richard Anthony, National Institute for Public Health and the Environment, BA Bilthoven, 3721BA, Netherlands, E-mail: richard.anthony@rivm.nl; Zhonghua Qin, Center for Accurate Detection of Tuberculosis, Tianjin Haihe Hospital, Tianjin, 300350, China. E-mail: 18920697956@189.cn

**Molecular Protocol for TB Pro assay**

**1 DNA Extraction**

In this study, we used **Nucleic Acid Extraction Reagent Kit** (YG-023-48) to extract nucleic acids.

**1.1 Sample Pretreatment**

Depending on the sample type, follow the sample pretreatment steps below:

1) Sputum Samples

a. Take 100 μL of sputum sample to a centrifuge tube, add 100 μL of 1 × PBS, vortex mix, briefly centrifuge, and incubate at 95°C in a constant-temperature mixer for 10 minutes. Cool to room temperature. Add 1 μL Internal Standard Working Solution of Internal Control.

b. Add 20 μL of Proteinase K, 200 μL of Lysis Buffer, and 1 μL of carrier RNA solution. Vortex and mix, briefly centrifuge, and incubate at 55°C in a constant-temperature mixer for 20 minutes at 300 rpm.

c. After incubation, briefly centrifuge, add 420 μL of Binding Buffer and 30 μL of MagBinding Beads. Vortex mix and place on a room-temperature 3D rotational mixer for 10 minutes. Proceed to "**1.2. DNA Extraction**" experiments.

2) Culture Samples

a. Culture medium treatment

1. Solid culture medium treatment: Pick colonies with an inoculating loop, add 300 μL of 1 × PBS to prepare a bacterial suspension.
2. Liquid culture medium treatment: Take 0.3~ 1 mL of liquid culture, centrifuge at 10,000× g for 15 minutes, discard the supernatant, and add 300 μL of 1×PBS to prepare a bacterial suspension.

b. Seal the tube tightly with a sealing film and incubate at 95°C in a constant-temperature mixer for 10 minutes. Add 1.5 μL Internal Standard Working Solution of Internal Control.

c. After incubation, centrifuge at 10,000×g for 1 minute, add 30 μL of Proteinase K, 300 μL of Lysis Buffer, and 1 μL of carrier RNA solution. Vortex mix, briefly centrifuge, and incubate at 55°C in a constant-temperature mixer for 20 minutes at 300 rpm.

d. After incubation, centrifuge at 10,000×g for 1 minute, collect 420 μL of supernatant in a new centrifuge tube, add 420 μL of Binding Buffer and 30 μL of MagBinding Beads. Vortex mix and place on a room-temperature 3D rotational mixer for 10 minutes. Proceed to "**1.2. DNA Extraction**" experiments.

**1.2. DNA Extraction**

1) Centrifuge the pre-processed samples briefly and place the centrifuge tube on a magnetic stand. Allow it to reset for 3 minutes until the MagBinding Beads are fully adsorbed, then carefully aspirate the supernatant.

2) Add 800 μL of Wash Buffer I to the centrifuge tube, vortex mix for 30 seconds to ensure the MagBinding Beads are in suspension, then place it in a temperature-controlled mixer at room temperature for 1 minute at 1,000 rpm.

3) After mixing and briefly centrifuge, place the centrifuge tube on a magnetic stand, and allow it to stand for 3 minutes until the MagBinding Beads are fully adsorbed. Aspirate the supernatant.

4) Add 800μL of Wash Buffer II to the centrifuge tube, vortex mix for 30 seconds to ensure the MagBinding Beads are in suspension, then place it in a temperature-controlled mixer at room temperature for 1 minute at 1,000 rpm. After mixing and briefly centrifuge, place the centrifuge tube on a magnetic stand, and allow it to stand for 3 minutes until the MagBinding Beads are fully adsorbed. Aspirate the supernatant.

5) Repeat step 4).

6) Remove the centrifuge tube from the magnetic stand, briefly centrifuge, then place it back on the magnetic stand to completely aspirate any remaining liquid inside the tube. Cover with the cap and incubate the centrifuge tube at 55°C for 1 minute in a temperature-controlled mixer.

7) After incubation, briefly centrifuge, add 40 μL of Nuclease-free Water to the centrifuge tube, vortex mix to ensure the MagBinding Beads are in suspension, and then briefly centrifuge. Place the tube in a temperature-controlled mixer at 55°C for 5 minutes at 1,000 rpm.

8) After incubation, briefly centrifuge the tube, place it on a magnetic stand, and let it stand for 2 minutes until the MagBinding Beads are fully adsorbed. Transfer 35 μL of the supernatant to a new centrifuge tube and label it to obtain the DNA solution.

**2 Library preparation**

**2.1 First round of amplification**

1) Take out the Multiplex PCR Mix and Multiplex PCR Primer Pool, thaw and place them on ice.

2) Invert the Multiplex PCR Mix upside down several times and briefly centrifuge; Vortex and briefly centrifuge Multiplex PCR Primer Pool. Prepare the amplification system according to Table 1, vortex, and briefly centrifuge.

**Table 1. First Round of Amplification Reaction System**

| Reagent | Volume (μL) |
| --- | --- |
| Template DNA | 20 |
| Multiplex PCR Mix | 9 |
| Total Volume | 29 |

1. Add Multiplex PCR Primer Pool to the the PCR tubes in the above reaction system. Use Nuclease-free Water to make the total volume of the reaction system 36 μL, vortex, and briefly centrifuge.
2. Place the prepared reaction system in the gene amplification instrument and start the program as outlined in Table 2.

**Table 2. First Round of Amplification Program**

| Temperature | Time | Cycles |
| --- | --- | --- |
| 37 ℃ | 10 min | 1 |
| 95 ℃ | 4 min | 1 |
| 95 ℃ | 30 s | 25 |
| 63 ℃ | 90 s |  |
| 72 ℃ | 10 s |  |
| 72 ℃ | 1 min | 1 |
| 4 ℃ | ∞ | — |

1. After PCR, place the PCR tubes at -25°C to -15°C for 2 minutes, then briefly centrifuge the tubes and proceed with **"2.2 First Purification"** experiment.

**2.2 First purification**

1) Before using the MagCleaning Beads in nucleic acid purification reagents, put it at room temperature for at least 30 minutes and thoroughly vortex it before use.

2) Prepare the first purification according to the Table 3, vortex,and briefly centrifuge. Leave at room temperature for use.

**Table 3. First purification system**

| Reagent | Volume (μL) |
| --- | --- |
| MagCleaning Beads | 40 |
| RNase-free Water | 14 |
| Total Volume | 54 |

1. Add first purification system reagents to the first round amplification products, vortex-mix, then incubate at room temperature for 5 minutes. After incubation, briefly centrifuge the PCR tube and place it on a magnetic rack.
2. After incubate at room temperature for 2 minutes, carefully remove the supernatant without disturbing the magnetic beads.
3. Remove the PCR tube from the magnetic rack,briefly centrifuge,and remove any remaining liquid from the bottom of the tube.
4. Add 20 μL of enzyme digestion reaction system reagents to the PCR tube (preparation see "**2.3 Enzyme digestion**"), remove the PCR tube from the magnetic rack, mix by vortexing, briefly centrifuge, and proceed to the next reaction.

**2.3 Enzyme digestion**

1) Take out the exonuclease, exonuclease buffer, RNase-free water, thaw and place on ice.

2) Vortex the exonuclease buffer and RNase-free water, briefly centrifuge; Invert the exonuclease upside down, and briefly centrifuge; The reaction system was prepared according to the Table 4, vortex, and briefly centrifuge.

**Table 4. Enzyme digestion Reaction System**

| Reagent | Volume (μL) |
| --- | --- |
| Exonuclease Buffer | 2 |
| Exonuclease | 1 |
| RNase-free Water | 17 |
| Total Volume | 20 |

3) Add enzyme digestion reaction system reagents to the first purification product (see "**2.2 First purification**"), vortex, and briefly centrifuge.

4) Place the prepared reaction system in the gene amplification instrument and start the program as outlined in Table 5 (reaction volume 20 μL).

**Table 5. Enzyme digestion Program**

| Temperature | Time | Cycle |
| --- | --- | --- |
| 37 ℃ | 15 min | 1 |
| 85 ℃ | 5 min | 1 |
| 4 ℃ | ∞ | — |

5) After the program ends, take out the enzymatic digestion product. Brief centrifuge,add the second round amplification reaction system to the PCR tube (prepared as described in "**2.4 Second Round Amplification**"), vortex, briefly centrifuge and proceed to the next step.

**2.4 Second Round Amplification**

1) Take out 2x Taq PCR Mix, Nuclease-free Water, dUTP, and Multiplex PCR Amplification Primer, thaw and place on ice.

2) Vortex and briefly centrifuge 2x Taq PCR Mix, Nuclease-free Water, dUTP, and Multiplex PCR Amplification Primer, prepare the second round amplification reaction system according to Table 6, vortex, and briefly centrifuge.

**Table 6. Second Round Amplification Reaction Program**

| Reagent | Volume (μL) |
| --- | --- |
| 2x Taq PCR Mix | 25 |
| dUTP (10 mM) | 1 |
| Total Volume | 26 |

3) Add 2μL different Multiplex PCR Amplification Primer to the PCR tube of the second round amplification reaction system,and record the identifier. Use nuclease-free water to make the total volume of the reaction system 30 μL.

4) Add the above reaction system to the enzyme digestion product (see "**2.3 Enzyme Digestion**"), vortex, and briefly centrifuge.

5) Place the prepared reaction system in the gene amplification instrument and start the program as outlined in Table 7 (reaction volume 50 μL).

**Table 7. Second Round Amplification Reaction Program**

| Temperature | Time | Cycles |
| --- | --- | --- |
| 94 ℃ | 4 min | 1 |
| 94 ℃ | 30 s | 15 |
| 60 ℃ | 30 s |  |
| 72 ℃ | 30 s |  |
| 72 ℃ | 1 min | 1 |
| 4 ℃ | ∞ |  |

6) After the program ends, take out the second round amplification product, briefly centrifuge, and proceed to "**2.5 Secondary Purification**" experiment.

**2.5 Secondary Purification**

1) Before using the MagCleaning Beads in nucleic acid purification reagents, put it at room temperature for at least 30 minutes and thoroughly vortex it before use.

2) Prepare fresh 80% ethanol.

3) Open the cap of the PCR tube containing the amplification product and add 40 μL of MagCleaning Beads. Cover with a cap, vortex-mix, and incubate at room temperature for 5 minutes.

4) After incubation, briefly centrifuge the PCR tube and place it on a magnetic rack for 3 minutes to allow the MagCleaning Beads to capture and clarify the solution. Discard the supernatant.

5) Add 180 μL of 80% ethanol at room temperature, let it sit for 30 seconds, then discard the supernatant. Cover with a cap.

6) Repeat step "e" .

7) Remove the PCR tube from the magnetic rack, briefly centrifuge for 3 seconds, and then place it back on the magnetic rack. Use a pipette to remove any remaining liquid from the bottom of the tube.

8) Add 30 μL of Nuclease-free Water for elution. Vortex thoroughly to completely resuspend the MagCleaning Beads. Incubate at room temperature for 5 minutes.

9) After incubation, briefly centrifuge the PCR tube and place it on the magnetic rack for 3 minutes. Once the MagCleaning Beads have captured and clarified the solution, transfer 27 μL of the supernatant to a new 1.5 mL centrifuge tube.

**3. Sequencing**

Sequencing can then be performed, by following instructions from Illumina platforms.
