# Supplementary figures and images for: A comprehensive evaluation of a novel targeted-sequencing workflow for Mycobacterium species identification and anti-tuberculosis drug-resistance detection
Source: Front Cell Infect Microbiol. 2025 Jun 9;15:1584237. doi: 10.3389/fcimb.2025.1584237 (PMC12183266; doi:10.3389/fcimb.2025.1584237)

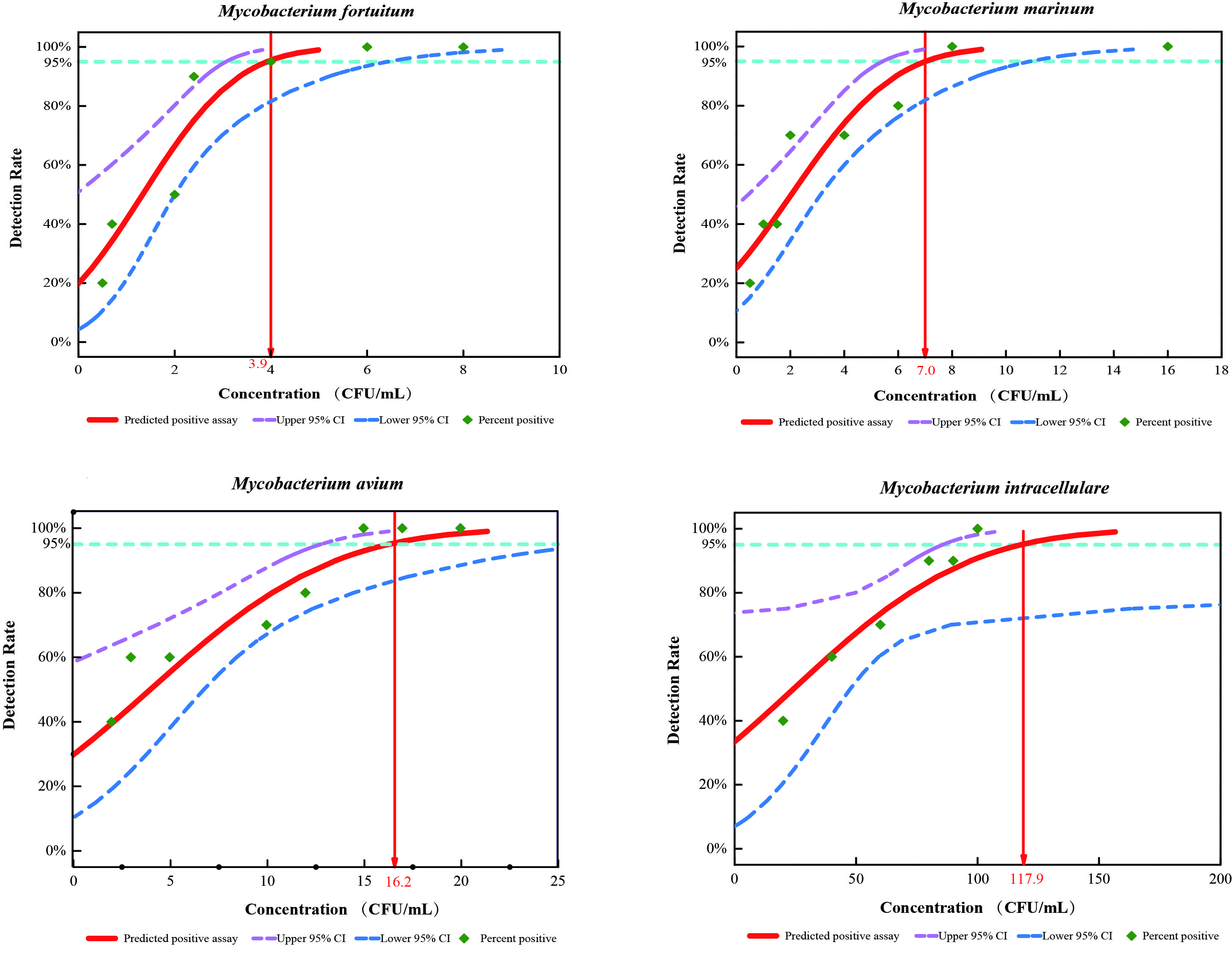

Supplement: Supplementary file 3 [file Image1.jpeg]

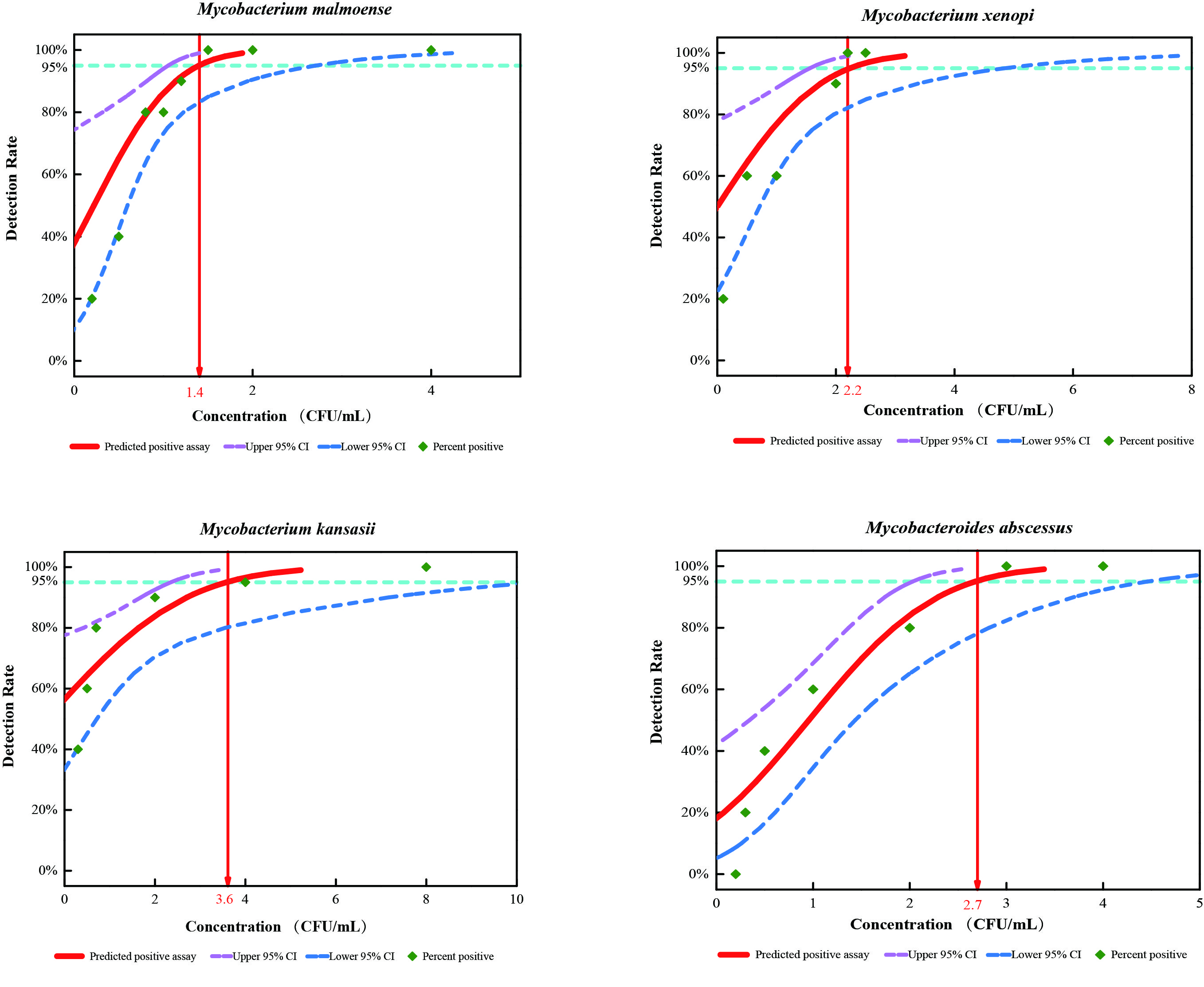

Supplement: Supplementary file 4 [file Image2.jpeg]
